# Acknowledgment

**Published:** 1905-02

**Authors:** 


### ACKNOWLEDGMENT.

Our readers from time to time have been, it is hoped, interested, and certainly instructed, in a series of papers read before the Section on Stomatology of the American Medical Association, at its last session, at Atlantic City, in June, 1904. We are indebted to the courtesy of the editor of the official journal of that body for the privilege of reproducing these valuable papers. It is thought that there has been very much gained by giving these articles a wider publicity in the pages of a journal more directly interested in the work of the Section originating them.

The series are now about concluded, and it is, therefore, deemed proper to give credit where credit is justly due. The thanks of the International Dental Journal are extended to the editor of the *Journal of the American Medical Association,* and also to the officers of the Section on Stomatology, for the privilege accorded.

